# Supplementary material for: Platelet-Derived Procoagulant Microvesicles Are Elevated in Patients with Retinal Vein Occlusion (RVO)
Source: J Clin Med. 2022 Aug 30;11(17):5099. doi: 10.3390/jcm11175099 (PMC9457368; doi:10.3390/jcm11175099)
Supplement: Supplementary file 1 [file jcm-11-05099-s001.zip › jcm-1881453-supplementary.pdf]

Platelet-Derived Procoagulant Microvesicles Are Elevated in Patients with Retinal Vein Occlusion (RVO)

Adrianna Marcinkowska[1,2], Nina Wolska[3], Boguslawa Luzak[1], Slawomir Cisiecki[2], Karol Marcinkowski[1] and Marcin Rozalski[1*]

Table S1. Platelet aggregation, platelet activation and reactivity markers in RVO patients and controls.

Parameter	RVO (n = 35)	CRVO (n = 21)	BRVO (n = 13)	Controls (n = 35)
**(A) Platelet activation markers ex vivo measured by flow cytometry**				
CD62 [%]	2.0 (1.5; 3.2)	2.0 (1.5; 3.2)	1.9 (1.3; 3.1)	1.7 (1.1; 2.5)
PAC-1 [%]	1.6 (0.9; 3.8)	1.2 (0.8; 2.7)	1.9 (1.0; 4.0)	1.2 (0.9; 3.9)
Fg binding [%]	4.2 (3.3; 5.2)	4.6 (4.1; 5.8)	3.4 (3.1; 5.1)	3.6 (2.7; 4.7)
Fg binding [MFI]	1289 (925; 1583)	1300 (988; 1462)	1261 (934; 51,827)	1363 (1007; 1544)
**(B) Platelet activation markers after stimulation with agonists in vitro measured by flow cytometry**				
CD62 ADP 1 µM [%]	22.0 (16.3; 33.9)	21.0 (16.3; 38.1)	22.0 (15.8; 28.1)	21.8 (12.0; 31.8)
PAC-1 ADP 1 µM [%]	11.9 (5.5; 20.7)	5.7 (2.9; 20.6)	16.2 (10.9; 26.8)	15.0 (7.6; 28.2)
Fg binding ADP 1 µM [%]	69.5 (46.7; 81.2)	57.6 (46.7; 85.9)	71.3 (67.5; 78.4)	67.1 (37.6; 79.7)
Fg binding ADP 1 µM [MFI]	3785 (2444; 5440)	3757 (2441; 5287)	3785 (2858; 5522)	3666 (2019; 4474)
CD62 ADP 10 µM [%]	48.6 (26.9; 61.9)	48.6 (31.0; 66.9)	46.6 (23.0; 54.3)	43.9 (30.4; 58.0)
PAC-1 ADP 10 µM [%]	40.2 (22.4; 62.1)	28.9 (22.4; 62.8)	49.1 (12.3; 62.1)	46.8 (26.0; 61.6)
Fg binding ADP 10 µM [%]	83.4 (74.5; 91.7)	79.4 (69.5; 92.4)	85.7 (76.6; 90.4)	85.9 (74.2; 89.9)
Fg binding ADP 10 µM [MFI]	5034 (3574; 7455)	5453 (3574; 6776)	4889 (4449; 7831)	4844 (3635; 6533)
CD62 col 5 µg/ml [%]	4.0 (2.9; 8.0)	3.3 (2.9; 9.3)	4.2 (3.5; 5.7)	4.2 (2.9; 6.9)
PAC-1 col 5 µg/ml [%]	4.1 (1.7; 8.7)	2.4 (1.3; 8.6)	5.2 (2.6; 8.6)	4.6 (2.6; 10.3)
Fg binding col 5 µg/ml [%]	10.5 (6.2; 22.5)	11.3 (4.8; 24.1)	10.6 (7.4; 12.3)	10.4 (6.4; 16.3)
Fg binding col 5 µg/ml [MFI]	2109 (1260; 2619)	1983 (1497; 2658)	2223 (1264; 2541)	2278 (1474; 2654)
CD62 col 20 µg/ml [%]	14.4 (5.8; 39.8)	7.1 (4.3; 48.6)	19.5 (11.0; 32.9)	18.1 (11.8; 34.9)
PAC-1 col 20 µg/ml [%]	17.3 (4.8; 38.2)	7 (3.8; 48.2)	26.4 (10.8; 35.0)	32.7 (12.4; 43.6)
Fg binding col 20 µg/ml [%]	29.0 (15.5; 57.7)	28.9 (12.4; 61.5)	19.8 (16.8; 57.4)	46.2 (25.3; 60.5)
Fg binding col 20 µg/ml [MFI]	2652 (1865; 3623)	2777 (1739; 3708)	2040 (1929; 3557)	2946 (2023; 4014)
CD62 TRAP 1 µM [%]	2.6 (2.1; 4.2)	2.5 (2.2; 4.3)	2.6 (2.0; 4.2)	2.3 (1.6; 3.7)
PAC-1 TRAP 1 µM [%]	1.7 (1.0; 3.5)	1.2 (0.8; 4.3)	2.4 (1.5; 3.4)	1.9 (1.1; 3.0)
Fg binding TRAP 1 µM [%]	6.8 (4.9; 11.1)	7.3 (5.8; 12.4)	5.3 (4.6; 10.1)	7.4 (5.2; 10.1)
Fg binding TRAP 1 µM [MFI]	1498 (1249; 2151)	1772 (1164; 2151)	1484 (1374; 2654)	1687 (1382; 2238)
CD62 TRAP 10 µM [%]	60.3 (38.1; 88.2)	61.7 (35.3; 89.4)	48.2 (46.6; 88.1)	82.3 (51.8; 90.2)
PAC-1 TRAP 10 µM [%]	36.3 (7.0; 56.1)	23.7 (4.9; 59.2)	45.4 (24.3; 56.0)	44.4 (21.9; 66.0)
Fg binding TRAP 10 µM [%]	57.7 (40.5; 92.0)	72.2 (37.9; 94.1)	53.6 (44.9; 82.2)	83.4 (70.6; 93.8)
Fg binding TRAP 10 µM [MFI]	5047 (2739; 6386)	5006 (2805; 7426)	5047 (2774; 5947)	5777 (4452; 6549)
**(C) Platelet aggregation measured in whole blood**				
AUC ADP 6.4 µM [au*min]	41.5 (30.5; 51.5)	40.5 (30.5; 62.8)	42.0 (33.6; 46.0)	51.0 (43.0; 58.0)
AUC Col 3.2 µg/ml [au*min]	75.0 (64.8; 82.3)	75.5 (64.0; 83.5)	72.0 (67.0; 79.0)	81.0 (68.0; 90.0)
AUC AA 0.5 mM[au*min]	51.0 (26.6; 59.8)	53.5 (29.5; 62.3)	46.0 (28.0; 58.0)	55.0 (40.0; 68.0)

Data presented as median as well as lower and upper quartile: Me (Q1; Q3). No statistically significant differences between RVO vs. controls, CRVO vs. controls, BRVO vs. controls, and CRVO vs. BRVO were found. Significance of differences between RVO and control group was estimated using Mann-Whitney U non-parametric test. Significance of differences between RVO groups and controls was estimated using Kruskal-Wallis non-parametric test with Dunn’s multiple comparison test. au—arbitrary units, AUC—Area Under the Curve, AA—arachidonic acid, Col—collagen, TRAP—Thrombin Receptor Activating Peptide, PAC-1—Platelet Activation Complex-1, BRVO—Branch Retinal Vein Occlusion, CRVO—Central Retinal Vein Occlusion, RVO—Retinal Vein Occlusion.
